# Prevalence and Predictors of Smoking among Gambian Men: A Cross-Sectional National WHO STEP Survey

**DOI:** 10.3390/ijerph16234719

**Published:** 2019-11-26

**Authors:** Bai Cham, Shaun Scholes, Nora E. Groce, Jennifer S. Mindell

**Affiliations:** 1 Medical Research Council Unit The Gambia at the London School of Hygiene and Tropical Medicine, Atlantic Road, Fajara, Banjul 273, The Gambia; 2 Department of Public Health, University of The Gambia, Brikama Campus, Brikama, Serrekunda 3530, The Gambia; 3 Research Department of Epidemiology and Public Health, University College London, London WC1E 6BT, UK; s.scholes@ucl.ac.uk (S.S.); nora.groce@ucl.ac.uk (N.E.G.); j.mindell@ucl.ac.uk (J.S.M.)

**Keywords:** tobacco use, smoking, The Gambia, WHO STEP survey

## Abstract

Background: Tobacco use is the leading cause of preventable death in the world, with a higher burden in low- and middle-income countries. The aim of this study was to quantify the prevalence and predictors of smoking among Gambian men using nationally representative data. Methods: Data was collected in 2010 from a random, nationally representative sample of 4111 adults aged 25–64 years (78% response rate) using the World Health Organization (WHO) STEPwise cross-sectional survey methods. Our analyses focused on men with valid information on smoking status (*n* = 1766) because of the low prevalence of smoking among women (1%). Results: The prevalence of current smoking among men was 31.4% (95% CI: 27.2–35.9). The median age of starting smoking was 19 years; 25% started before the age of 18 years and 10% started aged 8–10 years. Rural residence, underweight, and hypertension were significantly associated with smoking. Conclusion: The study reveals a high prevalence of smoking among Gambian men. It is evident that cigarettes are obtained by minors in The Gambia, as a high proportion of current smokers started at a young age. Advice and support to quit smoking should be extended to all smokers regardless of their age and whether or not they have any underlying health conditions.

## 1. Introduction 

Tobacco use is the leading cause of preventable death. Smoking is projected to increase to over one billion smokers globally by 2025 and a more rapid increase in the prevalence of smoking is predicted in Africa [1]. Tobacco companies are now shifting their target to low- and middle-income countries (LMICs) to build a broader consumer base [2,3]. The countries/regions with the highest levels of smoking are expected to shift from LMICs in Europe and the Western Pacific to Africa and the Eastern Mediterranean region [1]. The World Health Organization (WHO) Global Action Plan for the prevention and control of non-communicable diseases (NCDs) set up a number of global targets on the prevention and control of NCDs, including a 30% reduction in the prevalence of current tobacco use in persons aged 15 years and above by 2025. With the current trends [1], most of the countries in sub-Saharan Africa (SSA) will not meet this target. 

As evident in the literature, smoking is both a driver and a consequence of poverty in high-, middle- and low-income countries [4,5,6,7,8,9]. To satisfy the addictive nature of smoking, tobacco results in the diversion of income meant for important expenditures such as education and good nutrition. It can furthermore, lead to increased health care expenditure as a result of tobacco related health conditions [9]. 

Smoking is typically socially patterned. People living in more deprived areas are more likely to smoke. For example, in Ghana, smoking was more prevalent in those with lower socioeconomic status; smoking was also associated with a lower likelihood of purchasing health insurance [9]. It can also be argued that for some people, lack of education, limited resources, and ongoing poverty induces them to engage in risky behaviours including substance abuse and cigarette smoking [4,10,11]. Therefore, smoking contributes to poverty but poverty/lack of opportunity/lack of hope for a better future may also lead some people to take up or continue smoking. Smoking therefore, hinders the attainment of the United Nations Sustainable Development Goals, especially Goals: 1, which aims to end poverty; 3, which aims to ensure healthy lives and promote wellbeing; and 10, which aims to reduce inequality [12].

A limited number of studies on tobacco use have been conducted in The Gambia, with many of these being limited to youth and school children. A recent study among secondary school students (aged 12–20 years) demonstrated a high prevalence among students who had ever smoked (‘ever smoking’) constituting 26% and 9% for boys and girls, respectively [13]. An earlier survey among 13–15-year-old students reported a prevalence of ever smoking among boys and girls of 29% and 20%, respectively [14]. The 2018 Multiple Indicator Cluster Survey (MICs) in The Gambia found that 41.3% of men aged 15–49 years had ever used any tobacco products and that 18.9% had used it within the month prior to the survey [15]. Among women, the prevalence of ever and current use of cigarettes were 3.5% and 0.4%, respectively. The same survey reported that 4.9% of men and 1.0% of women had smoked before their 15^th^ birthday [15]. These results indicate that, despite laws in place banning the exposure of cigarettes to minors, cigarettes can be obtained by minors in The Gambia. The recent WHO NCDs country profile of The Gambia indicates that the prevalence of current smoking among adults aged 18 years and above was 15% based on projections [16]. A qualitative study conducted in 2016 on the implementation of the WHO Framework Convention on Tobacco Control (FCTC) found low awareness of the policies covered by the FCTC among policy makers in The Gambia [17]. A national survey on illicit trade in 2017 found that 7.3% of smokers had purchased an illicit cigarette at their last purchase [18].

Notably, most existing research on tobacco use at the national-level has been conducted in high income countries and is not always relevant to LMICs. We have not found a recent study on smoking among adults in The Gambia apart from the surveys mentioned above. An older study conducted in 1996–1997 among adults aged 15 years and above reported a smoking prevalence of 34% in urban areas and 42% in rural areas among men [19]. There is no up-to-date nationally representative study/data on the prevalence and associated risk factors of smoking among Gambian adults. Such evidence is needed to guide policy formulation and intervention strategies in tackling the burden of tobacco use. Data are also urgently needed to serve as a benchmark to monitor the prevalence of smoking in The Gambia over time, exposure of minors to tobacco, compliance with smoke free regulations, etc. The aim of this study was to quantify the prevalence and predictors of smoking among Gambian adults using nationally representative data. 

## 2. Materials and Methods

### 2.1. Materials 

This study is based on secondary analysis of data from the 2010 WHO STEP survey conducted among adults aged 25–64 years in The Gambia. This is the most recent nationally representative population-based health examination survey available. Data were collected from a random sample of adults aged 25–64 years from January to March 2010 using the WHO STEPwise approach [20,21]. The WHO STEPwise approach is a standard population-based health examination survey approach to NCD surveillance. It was initiated by the WHO in 2000 and is primarily conducted in LMICs [20,22,23,24]. Data are potentially collected in three main steps. Step one involves the use of interview-based questionnaires, where information on sociodemographic and key behavioural risk factors including age, gender, education, ethnicity, residence, tobacco use, alcohol consumption, fruit and vegetable consumption and physical inactivity are collected through face-to-face interviews. Information on previous history of diabetes and hypertension, and information on the treatment of these diseases is also collected. Physical measurements of weight, height, waist circumference and blood pressure are collected in step two. Step three involves biochemical measurement of blood glucose and cholesterol [20]. However, the STEP survey in The Gambia was limited to Steps one and two only. The interviews and physical measurements were conducted at respondents’ households [25].

Sample-selection weights and post-stratification weights were applied. This was done to account for differences in the probability of selection and also to adjust for differences between the age-gender distribution of the achieved sample and that of the target population. The 2003 population census of The Gambia (the most recent census prior to the time of data collection) was used as the target population to ensure the sample was nationally representative. We have compared the distribution of our data in terms of age, gender, and residence, with both the 2003 and 2013 census data and they are reassuringly very similar.

### 2.2. Dependent/Outcome Variables

The definition of the variables was based on the WHO STEP criteria. We used the standard questions below from the WHO STEP survey questionnaire (File S1) to measure the key variables. Current smoking status was captured by two questions:

1. “Do you currently smoke any tobacco products, such as cigarettes, cigars or pipes? (responses: Yes; No)”
2. “In the past, did you ever smoke daily? (responses: Yes; No)”

We used two variables on smoking: (1) a binary variable on current smoking status, categorised into not current (ex-smokers and never smokers) and current smokers, and (2) a derived variable with three categories (never, ex–smokers and current smokers). Ex-smokers were those participants who did not currently smoke but who had smoked daily in the past. 

We also assessed the mean age at which current smokers started to smoke. This was derived from the question: “How old were you when you first started smoking daily?”

### 2.3. Independent Covariates/Predictor Variables

The predictor variables included sociodemographic and behavioural risk factors including self-reported age, ethnicity, education, residence, fruit and vegetable intake, physical inactivity, objectively measured body mass index (BMI) and waist circumference, and hypertension. 

The eight ethnic groups in The Gambia were used (Mandinka, Fula, Wollof, Jola, Serahule, Serer, Manjago and Aku). We combined four minority groups (Serahule, Serer, Manjago and Aku) into a single ‘others’ category because of their small numbers. We derived an education variable based on the question “In total how many years have you spent at school or in full time study (excluding pre-school)?” and categorised the number of years into ≤ 6 y, 7–12 y and > 12 y.

Residence was determined by the respondents’ local government area (LGA) using the seven administrative regions in the country. We combined Banjul and Kanifing Municipality because of similar profiles (both urban). In addition, we explored differences by residence using a three-fold classification of urban, semi-urban, and rural based on The Gambia Bureau of Statistics benchmarks [26].

BMI and waist circumference were used to determine generalised and abdominal obesity, respectively. The WHO STEPS protocol requires objective physical measurements of height, weight, and waist circumference [25]. BMI (body weight in kilograms divided by height in metres squared: kg/m^2^) was categorised into underweight (BMI < 18.5 kg/m^2^), normal weight (18.5–24.9 kg/m^2^), overweight (25.0–29.9 kg/m^2^) and obese (BMI ≥ 30.0 kg/m^2^). Waist circumference was used to determine abdominal obesity based on the International Diabetes Federation thresholds (≥90 cm men; ≥80 cm women) [27]. 

Hypertension was defined as measured systolic blood pressure ≥ 140mmHg and/or diastolic blood pressure ≥ 90mmHg and/or self-reported hypertension (diagnosed by a doctor or other health professional, which included all participants reporting treatment).

Low fruit and vegetable intake were defined as consuming less than five combined servings (400 g) of fruit and vegetables a day. This is the minimum recommended by the joint World Health Organisation and the Food and Agricultural Organisation Expert Consultation on diet, nutrition and the prevention of chronic diseases and by the American Heart Association Nutrition Committee [28,29]. 

We assessed the level of physical activity to determine whether participants met the minimum WHO activity recommendations in a typical week [30]. Meeting the recommendations requires achieving at least 75 minutes/week of vigorous intensity physical activity, 150 minutes/week of moderate intensity activity, or a pro rata combination (i.e., achieving at least 600 metabolic equivalents (METS)/week). 

### 2.4. Statistical Analysis

We limited our analysis to men because of the low prevalence of smoking among adult women (1%). The analytical sample consisted of 1766 men with valid information on current smoking status. We report the prevalence of smoking in men in the form of proportions with corresponding 95% confidence intervals (95% CI). The prevalence estimates are weighted and adjusted for the complex survey design, including adjustment for non-response. We ran age-adjusted bivariate and fully-adjusted multivariate logistic regression models to identify factors associated with current smoking, comparing current smokers with those not currently smoking (never and ex-smokers). We also ran multinomial logistic regression analysis on smoking with three categories: never smoking, ex-smoking and current smoking, using never smoking as the reference group. Age-adjusted (aOR) and fully-adjusted odds ratios (AOR) with corresponding 95% CI are reported for the analysis of smoking using two categories, while age-adjusted (aRRR) and fully adjusted relative risk ratios (ARRR) are reported in the multinomial logistic regression models (smoking status represented by the three categories listed above). All analyses were weighted for non-response and adjusted for the complex survey design, using Stata V.15.0 (StataCorp LP, College Station, Texas, USA).

## 3. Results

### 3.1. Prevalence of Smoking among Men

**Figure 1:**
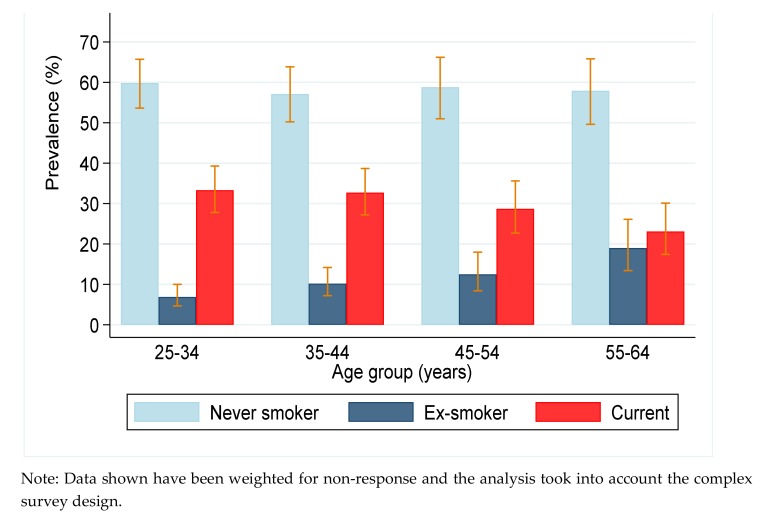
Prevalence of smoking in men by age.

**Figure 2:**
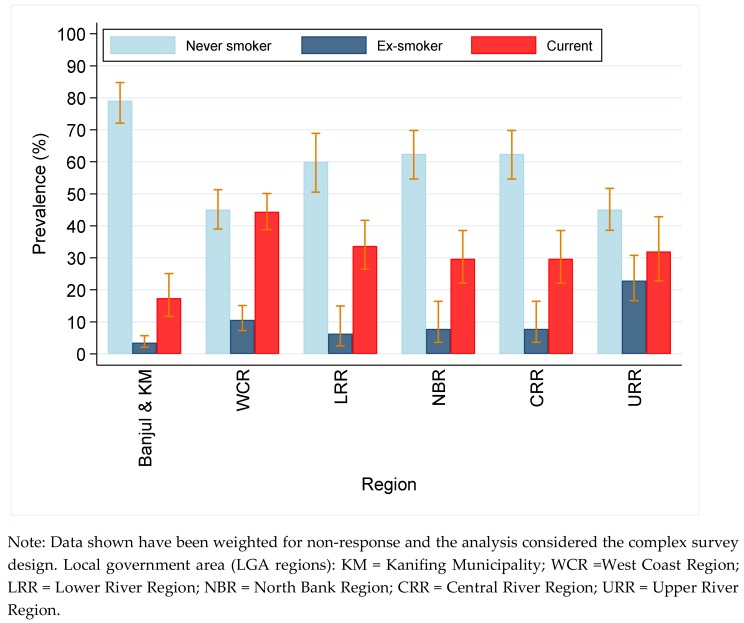
Prevalence of smoking in men by region.

The mean age of the male participants was 41 years; more than 60% were aged 25–44 years; and 62% had less than seven years of education (data not shown). The prevalence of smoking by age and region (LGA) of residence are presented in Figure 1 and Figure 2, respectively. The prevalence of current smoking among men was 31.4% (27.2–35.9). More than half of the men were never smokers and 10.0% (7.9–12.5) were ex-smokers (Table S1). The median age of starting smoking was 19 years; 25% started before the age of 18 years and 10% started aged 8–10 years. The average number of cigarettes smoked per day was 10 (95% CI: 9.1–10.8). The prevalence of current smoking was high in all age groups but it was significantly higher in younger men. However, the prevalence of ex-smoking was higher in the oldest age group (55–64 years). 

The prevalence of smoking was significantly higher in semi-urban and rural areas compared with urban areas. The prevalence of both current and ex-smoking were lowest in Banjul (the capital city) and Kanifing Municipality when we used region of residence (LGA) to distinguish between degrees of urbanicity (Figure 2). Both current and ex-smoking were significantly higher among the more physically active participants compared with the less active participants (see Table S1). However, this finding could be age-related as younger men were more physically active. The prevalence of current smoking was significantly higher among the underweight (47%, 95% CI: 39.0–54.8) and those with a normal weight (36%, 31.6–41.1) compared with the overweight (24%, 18.8–29.6) and the obese (24%, 15.7–33.8) (Table S1). 

The prevalence of ex-smoking was more than twice as high among hypertensives (17%, 12.0–22.6) compared with those normotensive (7%, 5.3–10.1). Based on a three-fold classification of hypertension status (normotensive, diagnosed, and undiagnosed hypertension), the prevalence of ex-smoking was more than twice as high among those who had their hypertension diagnosed by a doctor or other health professional (32%, CI: 21.3–43.8) compared with those who were undiagnosed (13%, CI: 8.3–20.0). 

### 3.2. Multivariate Regression Analysis of Factors Associated with Current and Ex-Smoking

We ran a multivariable binary logistic regression model comparing not current smokers (never and ex-smokers: coded 0) versus current smokers (coded 1). Only older age (55–64 years vs. 25–34 years (AOR 0.6, 95% CI: 0.4–0.9, P = 0.018)) and being overweight vs. normal weight (AOR 0.6, 0.4–0.8, P = 0.001) were significantly associated with the odds of current smoking (data not shown). 

We also ran a multivariable multinomial logistic regression model on the three-fold classification of current smoking status using never smokers as our reference (Table 1). Only overweight was significantly associated with the odds of current smoking versus never smoking. Compared with those with a normal weight, overweight persons were less likely to be current as opposed to never smokers (ARRR 0.5, 95% CI: 0.4–0.8). However, the odds of being an ex-smoker versus never smoker significantly increased with increasing age. Other factors significantly associated with being an ex-smoker versus never smoker were underweight vs normal weight (ARRR 2.0, 1.2-3.4), high waist circumference vs. normal/desirable waist circumference (ARRR 2.0, 1.3–3.0) and diagnosed hypertension vs normotensive (ARRR 2.6,1.1–6.2). 

## 4. Discussion

This analysis reveals a high prevalence of current smoking among Gambian men aged 25–64 years (31%). However, the prevalence of smoking among women was low (1%). The median age at which male participants who were current smokers started smoking was 19 years and many started when they were children. We have not found a recent study on smoking among adults in The Gambia in the literature but a study conducted between 1996 and 1997 among adults aged 15 years and above reported a smoking prevalence of 34% in urban areas and 42% in rural areas among men [19], compared with the prevalence of 29% in urban and 36% in rural areas among men in the 2010 STEPS survey data we observed in this analysis. The prevalence of smoking among Gambian men appears to have decreased over time, however we could not make direct comparisons because of the different age groups enrolled in the two studies. 

A recent nationwide study among secondary school students aged 12–20 years demonstrated a high prevalence of ever smoking (26% and 9% for boys and girls respectively) [13]. An earlier survey using the Global Youth Tobacco Survey methods conducted among students aged 13–15 years reported a prevalence of ever smoking to be 29% and 20% among boys and girls, respectively in The Gambia, whilst levels of current smoking were 13% and 9%, respectively [14]. The marked difference observed between the girls in that study and the women aged 25–64 years in our study could be because women in our study grew up at a time when girls did not smoke, as the prevalence of ex-smoking was less than 1%. These recent findings show that young Gambians, including girls, initiate smoking at an early age and are being exposed to smoking at a very young age. Generally, use of tobacco, especially smoking, continues to be less acceptable among females in SSA. However, this seems to be changing and requires further research. Certainly these recent findings among students show a shift towards increasing use among younger girls that is of concern [13].

Very few studies that use the WHO STEP approach explore factors associated with tobacco use in multivariable regression; three of these were conducted in Zambia [31,32,33], one in Benin [34], and another in South Africa [35]. In those countries, tobacco use was higher among males, rural residents, people from lower socio-economic backgrounds, and those with lower levels of education. Only being overweight (versus normal weight) was significantly associated with lower odds of current smoking (as opposed to never smoking) in our fully adjusted multinomial logistic regression models. However, being underweight (versus normal weight) and having been diagnosed with hypertension (versus normal blood pressure) were significantly positively associated with being an ex-smoker as opposed to never smoker. Research has shown, that both weight perception and actual weight are associated with smoking status [36,37]. People who do not believe smoking cigarettes help in maintaining normal body weight are less likely to report smoking. [36] The stronger association of ex-smoking versus never smoking amongst men with diagnosed hypertension compared with those normotensive and who had undiagnosed hypertension is not a surprising finding. It is likely that ex-smokers with diagnosed hypertension were advised to quit because of their health condition. The survey does not have information on whether current smokers have received any advice to stop smoking.

This study was conducted among adults (aged 25–64 years) but the results reveal that many current smokers started smoking as children or teenagers. There are a number of existing regulations and policies on tobacco control in The Gambia, including the Prohibition of Smoking in Public Places Act [38]. The Tobacco Control Act 2016, was adopted in December 2016 and it came into force in July 2018 [39]. The Tobacco Control Regulations 2019 [40], which supports the enforcement of the Tobacco Control Act 2016 has been signed and gazetted in August 2019. The country ratified the WHO Framework Convention on Tobacco Control (WHO FCTC) [41] in September 2007. There is also a ban on tobacco advertisements and promotion, as well as a ban on all tobacco-related sponsorship. In April 2016, the Ministry of Health of The Gambia launched a three-year national tobacco cessation clinical guideline with the objective of providing standardised treatment to tobacco users. The country has implemented a specific excise tax on all imported tobacco products, which has contributed to a price increase and reduction in the number of tobacco imports [42]. The government gained more revenues as a result of the increment in tax. Total importation of cigarettes decreased from 2012–2013 but tax collection from cigarettes doubled from GMD88.62 million to GMD166.90 million (US $1 = GMD33.34 in 2012) during this period [42]. Most of these initiatives were implemented after the data used for this research (2010) was collected. Therefore, we could not explore in this study whether the policies have contributed to a reduction in tobacco use, but it is an important subject for future research.

### 4.1. Policy Recommendations 

Our findings reveal that a high proportion of current smokers in The Gambia started smoking daily before their 18th birthday. Although our data was collected prior to the enactment of the Tobacco Control Act 2016, recent findings among secondary school students [13] and findings from the 2018 MICS [15], that focused on adults 15–49 years show that minors have access to tobacco. Article 16 of the WHO FCTC [41], the Gambia Tobacco Control Act 2016 [43] and the Tobacco Control Regulations 2019 [40] prohibit the sale of tobacco products to minors. These findings suggest that the implementation of the WHO FCTC and Tobacco Control Act are lacking in The Gambia, given that minors are successfully obtaining cigarettes. The risk of nicotine dependence is higher among smokers who start at an early age [44]. There is evidence in the literature suggesting that laws prohibiting illegal sales of cigarettes do not deter illegal sales and have limited influence of reducing prevalence of tobacco use if not enforced [2,45]. On the other hand, strict enforcement of regulations prohibiting sales of tobacco products to minors has been shown to contribute to a reduction of smoking among youths [45]. Research evidence has also shown that higher cigarette prices has decreased tobacco use onset among youths in Ghana and Nigeria [46], and this is reflected in the global literature as well. Based on these findings, the prices of cigarettes should be increased to deter youths from initiating tobacco use. Furthermore, the Government should put in place intervention strategies and regulations to reduce the exposure of young people to second hand smoke.

Although not substantiated by our data, as information on cessation support was not collected, research evidence has shown that support for young smokers who intend to quit smoking is very limited in The Gambia [13]. Smoking cessation support should be provided to all smokers, regardless of their age. Government, with support from its partners, should strongly regulate the exposure of minors to tobacco products, both because of the health risks of inhaling others’ smoke and the adverse effect of smoking role models on future smoking habits. Although some studies have shown that sensitisation reduces smoking intake among youths [47,48], other studies have revealed that targeting teenagers with non-smoking messages provided by the tobacco industry, influences—positively—attitudes towards the tobacco industry [49] and are at best, ineffective in deterring smoking uptake [50]. In summary, youth smoking is most effectively dealt with by preventing exposure to second-hand smoke and by a comprehensive raft of tobacco control policies and smoking cessation interventions aimed at adults [51,52]. Policies and regulations prohibiting smoking in public places should also be enforced. 

The prevalence of smoking was low among women aged 25–64 years in this study (1%). Our study was conducted among adults but recent findings among students aged 12–20 years shows a shift among girls and adolescent females, with 9% being current smokers [13]. With these findings, it can be anticipated that the levels of smoking among women will rise and this will be an important group to target for public health intervention efforts. Since taxes on tobacco products have proven to be effective in reducing consumption in The Gambia [42], this should be increased further. The proceeds from the increment in tax can be used to fund health promotion activities including smoking cessation programmes which could result in consequent health gains.

### 4.2. Strengths and Limitations of This Study

A main strength of this study is its novelty. There has only been a limited number of previous studies in The Gambia and these were largely conducted among youth and school children. This is the first nationally representative study on the prevalence and associated risk factors of smoking among Gambian men. It has identified key findings that could be used in policy formulation and intervention strategies in tackling the burden of tobacco use. It can also serve as a benchmark to monitor the prevalence of smoking in The Gambia over time.

A major limitation of this study is that our data are cross-sectional, which limits making causal inferences on the findings. Another limitation is the relatively small sample used for the analysis. We focused only on men because of the low prevalence of smoking among women. This might have limited the statistical power to detect any significant differences between groups. 

As described above, a number of tobacco control policies and initiatives have been implemented in The Gambia recently, including raising of the excise tax on all imported tobacco products, which has contributed to a price increase and reduction of tobacco imports [42]. However, these were implemented after the data used for this research was collected. Therefore, we could not explore if these policies and initiatives have affected levels of tobacco use. 

Another possible limitation is that the data on current smoking was self-reported, possibly biasing the prevalence estimates. Previous research has found significant under-reporting among participants who had their reported smoking status confirmed by taking measurements of their carbon monoxide level or concentration of serum/saliva cotinine [53,54]. Unlike other health surveys, such as the Health Survey for England, cotinine levels were not measured in our study, so we could not confirm the accuracy of participants’ reported smoking status. Women may also give a biased report of their smoking status for fear of stigmatisation, as smoking is socially undesirable among women in SSA [55]. Finally, no nationally representative study had been conducted among adults prior to this study to enable us to determine if the prevalence of smoking at the population level has been increasing or decreasing in The Gambia.

## 5. Conclusions

The study reveals a high prevalence of smoking among Gambian men, most of whom started smoking at a young age. Preventive efforts should be focused more on the younger age groups including children and young adults and the exposure of tobacco products to minors should be regulated. Advice and support to quit smoking should be extended to all smokers regardless of their age and whether or not they have any underlying health conditions.

## Figures and Tables

**Table 1:** Multinomial logistic regression on factors associated with smoking in men ^a,b^.

	Model I (Age Adjusted)		Model II (Fully Adjusted)	
	Ex-Smoker	Current-Smoker	Ex-Smoker	Current-Smoker
Variable	^a^ RRR(95% CI)	^a^ RRR(95% CI)	ARRR (95% CI)	ARRR (95% CI)
**Age Group**				
25–34	Reference	Reference	Reference	Reference
35–44	1.55(0.94–2.55)	1.03(0.77–1.38)	2.26(1.18–4.35) *	1.11(0.81–1.52)
45–54	1.84(1.11–3.05) *	0.88(0.60–1.28)	2.12(1.15–3.93) *	0.93(0.62–1.39)
55–64	2.84(1.69–4.78) ***	0.72(0.46–1.12)	3.76(1.79–7.87) ***	0.78(0.50–1.21)
**Ethnicity**				
Mandinka	Reference	Reference	Reference	Reference
Wollof	1.15(0.55–2.40)	0.62(0.39–1.02)	1.04(0.41–2.63)	0.65(0.38–1.11)
Fula	1.06(0.64–1.76)	1.26(0.84–1.90)	0.80(0.44–1.43)	1.14(0.73–1.76)
Jola	0.45(0.14–1.42)	1.13(0.76–1.70)	0.62(0.24–1.61)	1.07(0.68–1.68)
Others	0.57(0.25–1.29)	0.71(0.45–1.12)		0.63(0.36–1.12)
**Education**				
≤ 6 Years	Reference	Reference	Reference	Reference
7–12 Years	0.78(0.47–1.30)	0.74(0.50–1.09)	1.00(0.50–1.90)	0.81(0.53–1.23)
> 12 Years	0.51(0.21–1.22)	0.72(0.49–1.07)	0.61(0.25–1.51)	0.97(0.66–1.42)
**Residence** (Rurality)				
Urban	Reference	Reference	Reference	Reference
Semi urban	2.77(1.57–4.88) ***	1.28(0.76–2.15)	1.27(0.63–2.59)	0.91(0.53–1.56)
Rural	2.18(1.18–4.03) **	1.56(1.05–2.33) *	0.84(0.38–1.87)	0.92(0.62–1.37)
**Servings of fruits & vegs**				
≥ 5/day	Reference	Reference	Reference	Reference
< 5/day	1.11(0.55–2.26)	1.13(0.78–1.67)	0.99(0.47–2.04)	1.14(0.78–1.68)
**Physical activity**				
≥ 600METS/week	Reference	Reference	Reference	Reference
< 600METS/week	0.26(0.14–0.47) ***	0.26(0.13–0.51) ***	0.42(0.17–1.07)	0.77(0.42–1.43)
**BMI ^c^**				
Normal weight	Reference	Reference	Reference	Reference
Underweight	1.71(0.97–3.02)	1.71(1.18–2.48) ***	2.00(1.17–3.42) **	1.41(0.91–2.17)
Overweight	0.81(0.47–1.40)	0.53(0.38–0.74) **	0.60(0.30–1.17)	0.53(0.36–0.77) ***
Obese	0.58(0.26–1.32)	0.52(0.32–0.84) **	0.41(0.14–1.19)	0.64(0.35–1.17)
**Waist circumference ^d^**				
Normal	Reference	Reference	Reference	Reference
High	1.44(0.92–2.27)	0.74(0.42–1.26)	1.96(1.26–3.04) **	0.73(0.42–1.28)
**Hypertension ^e^**				
No (normotensive)	Reference	Reference	Reference	Reference
Yes (diagnosed)	3.99(1.94–8.20) ***	1.02(0.49–2.11)	2.64(1.12–6.23) ***	1.14(0.523–2.47)
Yes (undiagnosed)	1.61(0.86–3.02)	1.06(0.76–1.48)	1.76(0.88–3.51)	1.10(0.44–1.50)

Fully adjusted models: i.e., mutually adjusted for the variables shown in the table. ^a^ Data shown have been weighted for non-response and the analysis took into account the complex survey design. ^b^ Never smoking used as reference. ^c^ BMI is categorised into four groups as follows: underweight (BMI < 18.5kg/m^2^), normal-weight (18.5–24.9 kg/m^2^), overweight (25.0–29.9 kg/m^2^) and obese (BMI ≥ 30kg/m^2^). ^a^ RRR = Relative Risk Ratio adjusted for age (except for age group as the independent variable). ARRR= Adjusted Relative Risk Ratio(fully adjusted). * *p* < 0.05, ** *p* ≤ 0.01, *** *p* ≤ 0.001. METS = Metabolic equivalents. ^d^ Based on the definition of the International Diabetes Federation: High waist circumference, indicating abdominal obesity defined as ≥ 90cm in men and as ≥ 80cm in women. ^e^ Hypertension defined as measured SBP ≥ 140mmHg and/or DBP ≥ 90 mmHg and/or self-reported hypertension.

## Supplementary Materials

The following are available online at https://www.mdpi.com/1660-4601/16/23/4719/s1, Table S1: Prevalence of smoking in men by selected socio-demographic, behavioural and biological factors (*n* = 1766) a. File S1: WHO STEPS Instrument (Core and Expanded).

## References

[1] Bilano V., Gilmour S., Moffiet T., d’Espaignet E.T., Stevens G.A., Commar A., Tuyl F., Hudson I., Shibuya K. (2015). Global trends and projections for tobacco use, 1990–2025: An analysis of smoking indicators from the WHO Comprehensive Information Systems for Tobacco Control. Lancet.

[2] Chandora R., Song Y., Chaussard M., Palipudi K.M., Lee K.A., Ramanandraibe N., Asma S. (2016). Youth access to cigarettes in six sub-Saharan African countries. Prev. Med..

[3] WHO (2013). WHO Report on the Global Tobacco Epidemic, 2013: Enforcing Bans on Tobacco Advertising, Promotion and Sponsorship.

[4] Hiscock R., Bauld L., Amos A., Fidler J.A., Munafo M. (2012). Socioeconomic status and smoking: A review. Ann. N. Y. Acad. Sci..

[5] Belvin C., Britton J., Holmes J., Langley T. (2015). Parental smoking and child poverty in the UK: An analysis of national survey data. BMC Public Health.

[6] Xin Y., Qian J., Xu L., Tang S., Gao J., Critchley J.A. (2009). The impact of smoking and quitting on household expenditure patterns and medical care costs in China. Tob. Control.

[7] Wang H., Sindelar J.L., Busch S.H. (2006). The impact of tobacco expenditure on household consumption patterns in rural China. Soc. Sci. Med..

[8] John R.M., Sung H.Y., Max W.B., Ross H. (2011). Counting 15 million more poor in India, thanks to tobacco. Tob. Control.

[9] John R.M., Mamudu H.M., Liber A.C. (2012). Socioeconomic implications of tobacco use in Ghana. Nicotine Tob. Res..

[10] Haustein K.O. (2006). Smoking and poverty. Eur. J. Cardiovasc. Prev. Rehabil..

[11] WHO (2004). Tobacco and Poverty: A Vicious Circle.

[12] The United Nations (2015). Sustainable Development Goals.

[13] Jallow I.K., Britton J., Langley T. (2017). Prevalence and determinants of tobacco use among young people in The Gambia. BMJ Glob. Health.

[14] Manneh E. (2008). A Global Youth Tobacco Survey (GYTS) Country Report 2008.

[15] Gambia Bureau of Statistics (2019). The Gambia 2018 Multiple Indicator Cluster SurveySurvey Findings Report.

[16] (2018). World Health Organization—Noncommunicable Diseases (NCD) Country Profiles. https://www.who.int/nmh/publications/ncd-profiles-2018/en/.

[17] Jallow I.K., Britton J., Langley T. (2019). Exploration of policy makers’ views on the implementation of the Framework Convention on Tobacco Control in The Gambia: A qualitative study. Nicotine Tob. Res..

[18] Chisha Z., Janneh M.L., Ross H. (2019). Consumption of legal and illegal cigarettes in the Gambia. Tob. Control.

[19] Walraven G.E., Nyan O.A., Van Der Sande M.A., Banya W.A., Ceesay S.M., Milligan P.J., McAdam K.P. (2001). Asthma, smoking and chronic cough in rural and urban adult communities in The Gambia. Clin Exp Allergy.

[20] WHO (2003). STEPS: A framework for surveillance. The WHO STEPwise Approach to Surveillance of Non-Communicable Diseases (STEPS).

[21] Cham B., Scholes S., Ng Fat L., Badjie O., Mindell J.S. (2018). Burden of hypertension in The Gambia: Evidence from a national World Health Organization (WHO) STEP survey. Int. J. Epidemiol..

[22] WHO (2009). Ten years of the WHO STEPwise approach to chronic disease risk factor surveillance (STEPS): Challenges and opportunities (WHO Consultation). Review of International Experience in NCD Prevention & Control.

[23] Armstrong T., Bonita R. (2003). Capacity building for an integrated noncommunicable disease risk factor surveillance system in developing countries. Ethn. Dis..

[24] Riley L., Guthold R., Cowan M., Savin S., Bhatti L., Armstrong T., Bonita R. (2016). The World Health Organization STEPwise approach to noncommunicable disease risk-factor surveillance: Methods, challenges, and opportunities. Am. J. Public Health.

[25] WHO (2005). WHO STEPS surveillance manual. The WHO STEPwise Approach to Chronic Disease Risk Factor Surveillance.

[26] Gambia Bureau of Statistics (2013). The Gambia 2013 Population and Housing Census Preliminary Results.

[27] International Diabetes Federation (2006). The IDF Consensus Worldwide Definition of the Metabolic Syndrome.

[28] Joint WHO/FAO Expert Consultation (2003). Diet, Nutrition and the Prevention of Chronic Diseases.

[29] Lichtenstein A.H., Appel L.J., Brands M., Carnethon M., Daniels S., Franch H.A., Karanja N., Franklin B., Harris W.S., Lefevre M. (2006). Diet and lifestyle recommendations revision 2006: A scientific statement from the American Heart Association Nutrition Committee. Circulation.

[30] WHO (2012). Global Physical Activity Questionnaire (GPAQ) Analysis Guide.

[31] Siziya S., Babaniyi O., Songolo P., Nsakashalo-Senkwe M. (2011). Prevalence and correlates for tobacco smoking among persons aged 25 years or older in Lusaka urban district, Zambia. J. Public Health Epidemiol..

[32] Zyaambo C., Babaniyi O., Songolo P., Muula A.S., Rudatsikira E., Siziya S. (2013). Prevalence and predictors of smoking in a mining town in Kitwe, Zambia: A 2011 population-based survey. Health.

[33] Olusegun Babaniyi M.B.B.S., Muula A.S., Emmanuel Rudatsikira M.D. (2014). Prevalence and correlates for smoking among persons aged 25 years or older in two rural districts of Zambia. Int. J. Child. Health Hum. Dev..

[34] Houehanou Y.C., Lacroix P., Mizehoun G.C., Preux P.M., Marin B., Houinato D.S. (2015). Magnitude of cardiovascular risk factors in rural and urban areas in Benin: Findings from a nationwide STEPS survey. PLoS ONE.

[35] Maimela E., Alberts M., Modjadji S.E., Choma S.S., Dikotope S.A., Ntuli T.S., Van Geertruyden J.P. (2016). The Prevalence and Determinants of Chronic Non-Communicable Disease Risk Factors amongst Adults in the Dikgale Health Demographic and Surveillance System (HDSS) Site, Limpopo Province of South Africa. PLoS ONE.

[36] Coa K.I., Augustson E., Kaufman A. (2018). The Impact of Weight and Weight-Related Perceptions on Smoking Status Among Young Adults in a Text-Messaging Cessation Program. Nicotine Tob. Res..

[37] Jain P., Danaei G., Manson J.E., Robins J.M., Hernan M.A. (2019). Weight gain after smoking cessation and lifestyle strategies to reduce it. Epidemiology.

[38] The Government of The Gambia (1998). The Gambia Prohibation of smoking (Public Places) Act.

[39] Tobacco Conrol Laws: Legislation by Country. https://www.tobaccocontrollaws.org/legislation/country/gambia/summary.

[40] The Government of The Gambia (2019). Tobacco Control Regulations 2019.

[41] WHO (2003). Framework Convention on Tobacco Control.

[42] Nargis N., Manneh Y., Krubally B., Jobe B., Ouma A.E., Tcha-Kondor N., Blecher E.H. (2016). How effective has tobacco tax increase been in the Gambia? A case study of tobacco control. BMJ Open.

[43] The Government of The Gambia (2016). The Tobacco Control Act 2016.

[44] DeBry S.C., Tiffany S.T. (2008). Tobacco-Induced neurotoxicity of adolescent cognitive development (TINACD): A proposed model for the development of impulsivity in nicotine dependence. Nicotine Tob. Res..

[45] Stead L.F., Lancaster T. (2005). Interventions for preventing tobacco sales to minors. Cochrane Database Syst. Rev..

[46] Asare S., Stoklosa M., Drope J., Larsen A. (2019). Effects of Prices on Youth Cigarette Smoking and Tobacco Use Initiation in Ghana and Nigeria. Int. J. Environ. Res. Public Health.

[47] Brinn M.P., Carson K.V., Esterman A.J., Chang A.B., Smith B.J. (2010). Mass media interventions for preventing smoking in young people. Cochrane Database Syst. Rev..

[48] Wakefield M., Flay B., Nichter M., Giovino G. (2003). Effects of anti-smoking advertising on youth smoking: A review. J. Health Commun..

[49] Henriksen L., Dauphinee A.L., Wang Y., Fortmann S.P. (2006). Industry sponsored anti-smoking ads and adolescent reactance: Test of a boomerang effect. Tob. Control.

[50] Bates C., Watkins P., McNeill A. (2000). Danger: PR in the Playground: Tobacco Industry Initiatives on Youth Smoking.

[51] Lee K.A., Palipudi K.M., English L.M., Ramanandraibe N., Asma S. (2016). Secondhand smoke exposure and susceptibility to initiating cigarette smoking among never-smoking students in selected African countries: Findings from the Global Youth Tobacco Survey. Prev. Med..

[52] Albers A.B., Biener L., Siegel M., Cheng D.M., Rigotti N. (2008). Household smoking bans and adolescent antismoking attitudes and smoking initiation: Findings from a longitudinal study of a Massachusetts youth cohort. Am. J. Public Health.

[53] Hald J., Overgaard J., Grau C. (2003). Evaluation of objective measures of smoking status—A prospective clinical study in a group of head and neck cancer patients treated with radiotherapy. Acta Oncol..

[54] Brathwaite R., Addo J., Smeeth L., Lock K. (2015). A Systematic Review of Tobacco Smoking Prevalence and Description of Tobacco Control Strategies in Sub-Saharan African Countries; 2007 to 2014. PLoS ONE.

[55] Addo J., Smeeth L., Leon D.A. (2009). Smoking patterns in Ghanaian civil servants: Changes over three decades. Int. J. Environ. Res. Public Health.

