# Supplementary material for: Prevalence and Predictors of Smoking among Gambian Men: A Cross-Sectional National WHO STEP Survey
Source: Int J Environ Res Public Health. 2019 Nov 26;16(23):4719. doi: 10.3390/ijerph16234719 (PMC6926921; doi:10.3390/ijerph16234719)
Supplement: Supplementary file 1 [file ijerph-16-04719-s001.pdf]

Supplementary Table 1: Prevalence of smoking in men by selected socio-demographic, behavioural and biological factors (n=1766) [a]

Variable	Never smoker %(95% CI)	Ex-smoker %(95% CI)	Current smoker %(95% CI)	χ^2 P value
**Total**	58.7(53.5-63.7)	10.0(7.9-12.5)	31.4(27.2-35.9)	
**Age Group**				
25-34	59.8(53.6-65.7)	6.9(4.7-10.0)	33.3(27.8-39.3)	**0.001**
35-44	57.1(50.2-63.8)	10.2(7.2-14.2)	32.7(27.2-38.7)	
45-54	58.8(51.0-66.2)	12.5(8.4-18.0)	28.7(22.7-35.6)	
55-64	57.9(49.6-65.8)	19.0(13.4-26.1)	23.1(17.4-30.1)	
**Marital status**				
Never married	62.6(54.3-70.3)	5.4(2.8-10.0)	32.0(24.4-40.8)	0.072
Married	58.0(52.1-63.7)	11.9(9.3-15.2)	30.1(25.5-35.1)	
Separated	49.0(32.7-65.5)	11.8(5.0-25.3)	39.2(25.0-55.5)	
Widowed				
Cohabiting	54.7(45.6-63.6)	5.6(2.5-12.2)	39.7(31.9-48.0)	
**Occupation**				
Employed	59.5(51.7-66.8)	8.9(5.7-13.5)	31.7(25.3-38.8)	**<0.001**
Self employed	55.0(49.4-60.5)	11.1(8.5-14.4)	33.9(29.1-39.1)	
Non paid	58.2(41.6-73.0)	13.8(5.0-32.9)	28.0(17.6-41.6)	
Student	74.1(59.0-85.0)	5.2(1.3-18.2)	20.8(10.3-37.5)	
Housemaker	93.8(74.1-98.8)	4.8(0.6-28.1)	1.4(0.2-9.7)	
Retired	71.1(50.9-85.4)	26.3(13.2-45.7)	2.6(0.3-17.6)	
Unemployed	64.1(49.6-76.4)	1.9(0.6-5.4)	34.1(22.3-48.2)	
**Ethnicity**				
Mandinka	57.8(51.2-64.1)	10.3(7.4-14.2)	31.9(26.4-38.0)	0.052
Wollof	64.1(55.1-72.2)	13.9(8.1-22.6)	22.0(15.6-30.3)	
Fula	53.0(44.6-61.3)	10.1(7.2-14.1)	36.9(29.6-44.9)	
Jola	58.6(49.2-67.4)	5.0(1.9-12.7)	36.4(28.9-44.6)	
Others	66.6(56.0-75.80	7.4(3.9-13.5)	26.0(18.3-35.6)	
**Residence** (Local government area) [b]				
Banjul & KM	79.1(72.1-84.8)	3.5(2.1-5.7)	17.4(11.7-25.1)	**<0.001**
WCR	45.1(39.0-51.3)	10.6(7.3-15.1)	44.4(38.8-50.1)	
LRR	60.0(50.5-68.9)	6.3(2.5-15.0)	33.7(26.5-41.7)	
NBR	62.5(54.6-69.8)	7.8(3.6-16.4)	29.7(22.1-38.5)	
CRR	62.5(54.6-69.8)	7.8(3.6-16.4)	29.7(22.1-38.5)	
URR	45.1(38.6-51.7)	22.9(16.6-30.8)	32.0(22.8-42.8)	
**Residence** (Rurality)				
Urban	63.7(55.9-70.8)	7.3(5.2-10.2)	29.0(22.8-36.2)	**0.004**
Semi urban	52.7(45.8-59.5)	16.4(11.8-22.4)	30.9(23.2-39.8)	
Rural	51.1(45.1-57.1)	13.2(9.1-18.7)	35.7(31.0-40.8)	
**Education level**				
No formal education	58.1(52.3-63.7)	11.5(8.8-15.1)	30.4(25.4-35.9)	0.230
Primary/ Middle	54.9(45.6-64.0)	8.2(5.1-12.9)	36.9(29.3-45.2)	
Secondary/Tertiary	62.2(55.6-68.4)	8.5(5.5-13.0)	29.3(23.6-35.7)	
**Years spent in school**				
≤6 Years	56.6(50.8-62.1)	10.8(8.3-13.9)	32.6(27.8-37.9)	0.677
7-12 Years	60.8(53.6-67.6)	9.5(6.3-14.1)	29.7(23.5-36.7)	
>12 Years	59.9(50.2-68.9)	7.6(3.8-14.9)	32.5(24.8-41.2)	
**Servings of fruit and vegetables**				
≥ 5/day	60.5(50.6-69.6)	8.2(4.8-13.8)	31.3(23.8-39.8)	0.804
< 5/day	57.6(52.7-62.3)	9.0(6.6-12.3)	33.4(29.3-37.7)	
**Physical activity**				
≥600METS/week	55.6(51.2-60.0)	10.8(8.4-13.8)	33.6(29.9-37.5)	**<0.001**

Variable	Never smoker %(95% CI)	Ex-smoker %(95% CI)	Current smoker %(95% CI)	χ^2 P value
<600METS/week	81.9(72.2-88.8)	5.2(2.9-8.9)	12.9(7.0-22.7)	
**BMI**[c]				
Normal/desirable	54.2(48.8-59.4)	9.7(7.2-12.9)	36.2(31.6-41.1)	**<0.001**
Underweight	41.3(33.2-49.9)	11.9(7.5-18.2)	46.8(39.0-54.8)	
Overweight	66.3(59.9-72.1)	9.9(6.4-15.2)	23.8(18.8-29.6)	
Obese	68.9(57.6-78.3)	7.6(3.6-15.4)	23.5(15.7-33.8)	
**Waist circumference** [d]				
Normal	56.8(51.9-61.6)	8.9(6.9-11.5)	34.3(30.3-38.5)	0.057
High	59.2(48.7-69.0)	15.0(10.2-21.6)	25.8(16.9-37.3)	
**Waist-hip ratio** [e]				
Normal	58.6(53.5-63.4)	9.3(7.1-12.1)	32.2(28.1-36.5)	0.068
High	49.0(40.9-57.1)	11.0(7.7-15.6)	40.0(31.5-49.1)	
**Waist-height ratio**				
Normal (≤0.5)	56.0(50.7-61.1)	8.9(6.8-11.6)	35.1(30.8-39.7)	0.060
High (>0.5)	60.7(52.4-68.5)	12.99.0-18.3)	26.3(19.3-34.9)	
**Hypertensive** [f]				
No	59.6(54.2-64.8)	7.3(5.3-10.1)	33.1(28.6-37.8)	**<0.001**
Yes	52.0(44.4-59.6)	16.6(12.0-22.6)	31.4(26.0-37.3)	
**Hypertension**				
No (normotensive)	59.6(54.2-64.8)	7.3(5.3-10.1)	33.1(28.6-37.8)	**<0.001**
Yes (diagnosed)	39.3(28.3-51.5)	31.5(21.3-43.8)	29.2(18.5-42.9)	
Yes (undiagnosed)	55.0(46.4-63.4)	13.1(8.3-20.0)	31.9(25.9-38.6)	

[a] Data shown have been weighted for non-response and the analysis took into account the complex survey design.

[b] KM=Kanifing Municipality; WCR =West Coast Region; LRR= Lower River Region; NBR =North Bank Region; CRR = Central River Region; URR =Upper River Region

[c] BMI is categorised into underweight (BMI<18.5Kg/m^2), normal (18.5-24.9 Kg/m^2), overweight (25.0-29.9Kg/m^2) and obese (BMI ≥30Kg/m^2).

[d] Based on the definition of the International Diabetes Federation (High waist circumference, indicating abdominal obesity defined as ≥90 cm in men)

[e] Based on WHO standards (high waist-hip ratio defined as ≥90cm in men)

[f] Hypertension defined as measured SBP≥140mmHg and/or DBP and/or self-reported hypertension
